# Immune Modulatory Oxysterols Produced from Cholesterol-Containing Lipid Nanoparticles Regulate Tumor Growth

**DOI:** 10.1021/acsnano.5c22020

**Published:** 2026-03-13

**Authors:** Patricia Ines Back, Shadan Modaresahmadi, Jalpa Patel, Indhumathy Subramaniyan, Vindhya Edpuganti, Md. Rakibul Islam, Seonjuti Chowdhury, Adam J. Carr, Qisheng Zhang, William C. Putnam, Li Li, Ninh M. La-Beck

**Affiliations:** † Department of Immunotherapeutics and Biotechnology, Jerry H. Hodge School of Pharmacy, 385388 Texas Tech University Health Sciences Center, Abilene, Texas 79601, United States; ‡ Clinical Pharmacology and Experimental Therapeutics Center, Jerry H. Hodge School of Pharmacy, 15496 Texas Tech University Health Sciences Center, Dallas, Texas 75235, United States; § Department of Pharmacy Practice, Jerry H. Hodge School of Pharmacy, Texas Tech University Health Sciences Center, Dallas, Texas 75235, United States; ∥ Division of Chemical Biology and Medicinal Chemistry, Eshelman School of Pharmacy, 2331 University of North Carolina at Chapel Hill, Chapel Hill, North Carolina 27599, United States

**Keywords:** lipid nanoparticles, liposomes, cholesterol metabolism, oxysterols, cancer, macrophages, immunomodulation

## Abstract

Cholesterol is a component of most clinically approved lipid nanoparticles (LNPs) to enhance the stability, fluidity, and organization of the lipid structure. Endogenous cholesterol undergoes oxidation, producing cholesterol oxidation products (oxysterols), which are potent regulators of cellular processes implicated in immune dysfunction and the pathogenesis of cancers. LNP-associated cholesterol (LNP-cholesterol) is internalized by macrophages, and these cells also play critical roles in cholesterol metabolism and regulation of immune responses against cancer. Yet, the metabolic fate of LNP-cholesterol remains unclear. In this work, we elucidated the in vivo metabolic fate of LNP-cholesterol and demonstrated that LNP-oxysterols affect tumor cell proliferation and modulate macrophage functionality, impacting tumor growth in a murine model of cancer. Importantly, we showed that LNP-associated 7α-hydroxycholesterol, 7β-hydroxycholesterol, 24-hydroxycholesterol, and 27-hydroxycholesterol have antitumoral effects, while LNP-associated 7-ketocholesterol and 5,6-epoxycholesterol have protumoral effects, suggesting that cholesterol metabolism and cholesterol analogs can be leveraged to enhance LNP drug efficacy in cancer. Our findings indicate that LNP carriers have an unintended impact on tumor growth, which has the potential to diminish or enhance the anticancer efficacy of LNP-associated therapeutic cargo. Our results highlight the potential to engineer LNP carriers with immune-modulatory activity that combines the advantages of both nanoparticle-mediated drug delivery and immunotherapy.

## Graphical abstract

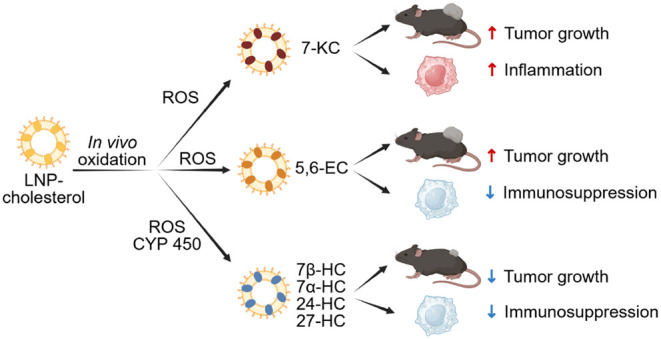

Lipid nanoparticles (LNPs) are widely used as carriers for the delivery of small molecules, nucleic acids, and proteins to increase drug bioavailability and stability and to achieve sustained drug release.^1^ In cancer drug delivery, LNPs reduce the toxicity of cytotoxic drug cargo and exploit the dysfunctional tumor vasculature to enhance tumor drug accumulation. In vaccine delivery, LNPs not only protect cargos, such as mRNA, from degradation but also enable intracellular delivery of nucleic acids. LNPs have been in clinical use for over 30 years,^2^ and their in vivo biocompatibility has led to the perception that they are biologically inert. However, compared to the conventional “free” form, the LNP form of a drug has increased interactions with the immune system.^3–5^ LNPs are internalized by phagocytic immune cells, such as macrophages, and can activate circulating immune proteins such as complement proteins.^6–9^ Blood complement activation by LNP-doxorubicin is implicated as a cause of acute infusion reactions in cancer patients,^10^ and peripheral blood monocyte count and phagocytic function correlate with LNP clearance rates in patients.^11,12^ This demonstrates that interactions of LNPs with the immune system affect drug tolerability and pharmacokinetics. Unfortunately, the impact of these interactions on the tumor immunologic milieu is largely unknown due to a paucity of systematic studies to assess the immune-modulatory activity of the LNP carrier.^13^

Cholesterol is a common component of LNPs that is used to enhance stability, fluidity, and organization of the lipid structure.^14–17^ Endogenous cholesterol undergoes both enzymatic and nonenzymatic oxidation, producing cholesterol oxidation products (i.e., oxysterols) that are potent regulators of cellular processes implicated in the pathogenesis of atherosclerosis, Alzheimer’s disease, and many cancers.^18^ The oxysterol 27-hydroxycholesterol (27-HC) can modulate immune responses by binding Toll-like receptors (TLRs) such as TLR4 on macrophages^19^ and inducing the production of IL-8,^20^ which has been linked to downstream activation of the CXCR2 pathway and tumor proliferation.^20,21^ Endogenous cholesterol is transported by lipoproteins, such as high-density lipoprotein (HDL) and low-density lipoprotein (LDL), via processes mediated by macrophages. Macrophages express a variety of receptors for cholesterol internalization, including scavenger receptors SR-A1, SR-E1 (LOX1), cluster of differentiation 36 (CD36), lipoprotein receptors (LDLR, VLDLR), and ApoE receptors (ApoER2),^1^ and they also express high levels of hydroxylases and reactive oxygen species (ROS).^1^ Oxysterols in circulating LDL-cholesterol induce macrophages to become dysfunctional foam cells,^22,23^ and several oxysterols (27-HC, 7-KC, 7α-HC, and 7β-HC) have been found in these cells.^24^ Importantly, LNP-associated cholesterol (LNP-cholesterol) has been shown to be internalized by macrophages through interactions with CD36, LDLR, and SR-A1 receptors,^25^ strongly suggesting that LNP-cholesterol may similarly generate oxysterols and induce macrophage dysfunction. Yet, the in vivo metabolic fate of LNP-cholesterol remains unclear.

Alterations in the sterol lipids not only modify cellular uptake of LNPs but can also impact protein expression and cellular functions, such as antigen presentation and immune activation, which have major implications for LNP-based vaccine development. For instance, β-sitosterol has anti-inflammatory properties^26^ and decreases CD8^+^ T cell responses when incorporated into LNPs.^27^ The addition of a fifth ring (e.g., cyclopentyl or cyclohexyl) in the cholesterol tail leads to steric hindrance, modifying the organization of the lipid bilayer and mRNA cargo and leading to a low encapsulation rate and transfection efficiency.^28^ Complete or partial replacement of cholesterol by bile acids (e.g., cholic acid, chenodeoxycholic acid, deoxycholic acid, and lithocholic acid) leads to increased extrahepatic tropism,^29^ while the replacement of cholesterol with cholic acid increases LNP tropism to the spleen.^29^ Nonetheless, how LNP-associated oxysterols affect vaccine efficacy has not been systematically studied.

This gap in understanding the metabolic fate of LNP-cholesterol has particular significance for cancer drug delivery because macrophages also play a pivotal role in regulating antitumor immune responses, and dysfunctional M2-like macrophages produce immunosuppressive cytokines/chemokines and protumoral growth factors that mediate cancer immune evasion.^30^ Since the majority of clinically approved LNPs are liposomes containing cholesterol, it is imperative to understand whether LNP-cholesterol undergoes in vivo metabolism and how LNP-associated oxysterols (LNP-oxysterols) can impact anticancer efficacy. Herein, we elucidate the in vivo metabolic fate of LNP-cholesterol and demonstrate that LNP-oxysterols affect tumor cell proliferation and modulate macrophage functionality, affecting tumor growth in a murine model of cancer. Importantly, we show that LNP-associated 7α-HC, 7β-HC, 24-HC, and 27-HC have antitumoral effects, while LNP-associated 7-KC and 5,6-EC have protumoral effects, suggesting that cholesterol metabolism and cholesterol analogs can be leveraged to enhance LNP drug efficacy in cancer.

## Results

### LNP-Cholesterol Undergoes Metabolism In Vivo through Enzymatic and Auto-oxidation Pathways

Systemically administered LNPs are known to accumulate in the liver and spleen, but whether they undergo metabolism in these and other tissues is unknown. To elucidate this, we first synthesized liposomes containing deuterated cholesterol (LNP-cholesterol-d7) to enable the separation of liposome-derived cholesterol and oxysterols from endogenous cholesterol and oxysterols. These liposomes were monodispersed (PDI 0.03) and similar to the liposome carrier for pegylated liposomal doxorubicin (PLD) in size (∼92 nm), zeta potential (−35 mV), and composition (40:55:5 molar ratio of cholesterol, HSPC, and mPEG_2000_DSPE) (Supplemental Table 1). We chose this model carrier since it is the most extensively used LNP formulation in cancer patients.^2^ These LNPs were then administered to wild-type C57Bl/6 mice by tail vein injection at a dose of 100.5 nmol/g of cholesterol-d7, which corresponds to the estimated human–mouse equivalent dose of cholesterol that is in 60 mg/m^2^ of PLD used in cancer patients.^31,32^ Mice were euthanized 24 h after dosing; blood and tissues were collected for LC–MS/MS quantitation of enzymatic (24-HC, 25-HC, 27-HC) and auto-oxidation (5,6-EC, 7-KC, 7α-HC, 7β-HC) cholesterol metabolites. Our results showed that LNP-derived cholesterol was found in the liver, spleen, lungs, heart, kidney, and plasma, with the highest amount of cholesterol-d7 and oxysterols found in the liver (Figure 1A and B), consistent with the role of the liver as a key organ in cholesterol transport and metabolism. In the liver, the predominant LNP-derived oxysterols were the enzymatic metabolites 24-HC and 27-HC (Figure 1C). Hepatocytes, sinusoidal endothelial, stellate, and Kupffer cells in the liver are the primary source of CYP27A1, which oxidizes cholesterol into 27-HC,^33^ while 24-HC is produced by CYP46A1, which is believed to be exclusively expressed by brain neurons.^34^ The presence of LNP-derived 24-HC suggests that there is LNP transport to the central nervous system with subsequent metabolism of LNP-cholesterol to 24-HC and transport to the liver, although extracerebral sources of 24S-HC have been reported.^35^ In contrast, the predominant oxysterols in the kidneys, spleen, lungs, heart, and plasma were 5,6-EC, 7-KC, and 7β-HC (Figure 1D–H), which are auto-oxidation metabolites of cholesterol formed through free radical-mediated oxidation. These organs, but not plasma, also have LNP-derived 24-HC and 7α-HC (Figure 1D–H). 7α-HC has been found in commercial LNPs and food products as products of auto-oxidation;^36,37^ however, it can also be generated by cholesterol 7α-hydroxylase (CYP7A1) in hepatocytes.^38^ Another likely source of these oxysterols is tissue macrophages, which produce large amounts of reactive oxygen species (ROS) when activated and can mediate the auto-oxidation of cholesterol.^39–41^

**Figure 1:**
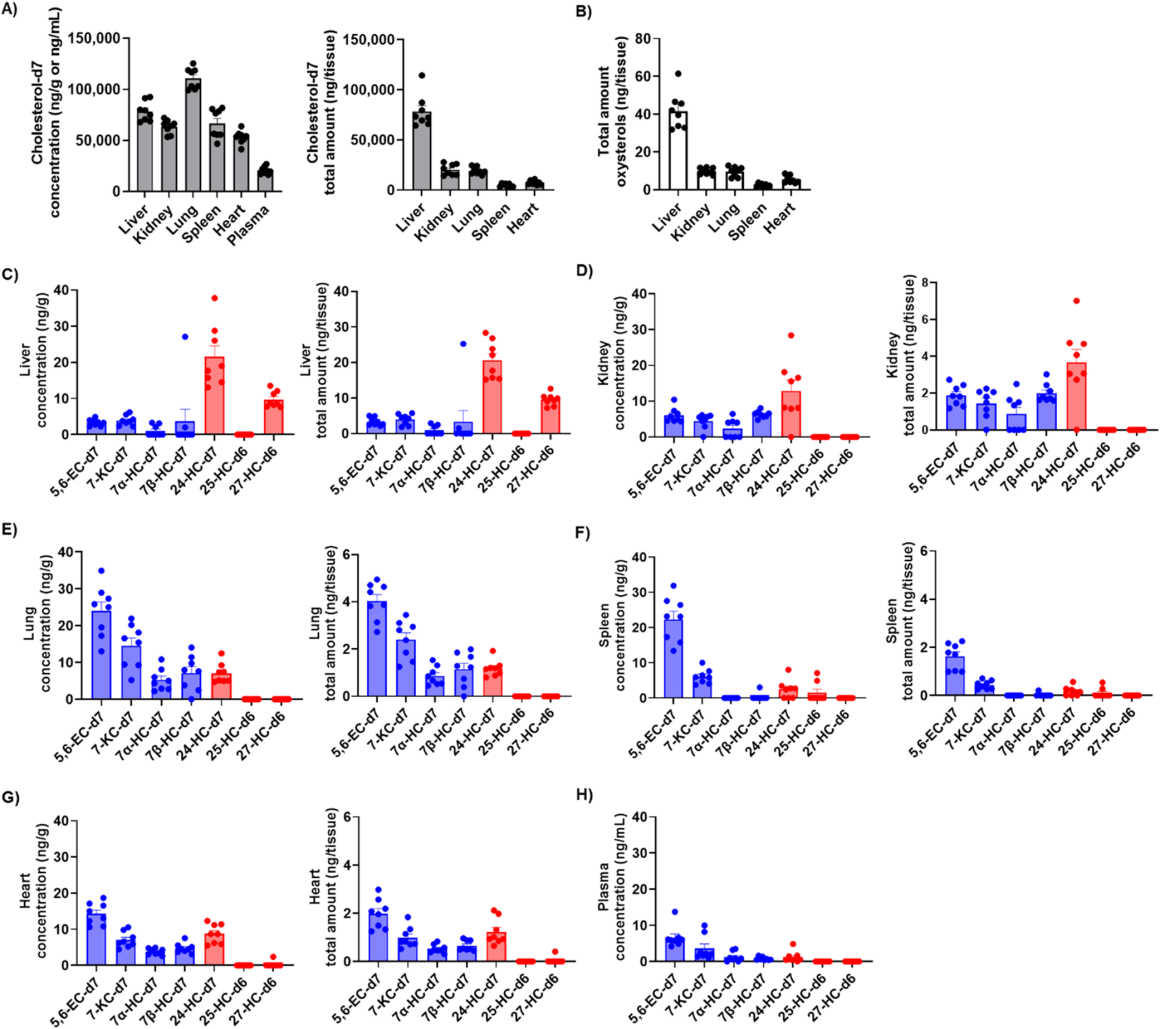
Quantification of deuterated cholesterol and deuterated oxysterols in tissues of C57BL/6 mice treated with LNP-cholesterol-d7. A) LNP-derived cholesterol concentrations and total amounts in tissues and plasma. B) Total amount of LNP-derived oxysterols in tissues. C–H) Tissue concentrations and total amount of LNP-derived oxysterols produced by autoxidation (blue) and enzymatic pathways (red). Each data point represents an individual mouse. Data represent the mean + SEM.

### In Vitro Macrophage LNP-Cholesterol Metabolism Occurs through Auto-oxidation

Given that LNPs are readily engulfed by macrophages,^1^ the intracellular trafficking of LNPs to subcellular compartments has been well studied, but whether LNP-cholesterol undergoes metabolism in macrophages has not been elucidated. Macrophages are typically activated in response to pathogens and tissue damage; however, LNPs have also been reported to induce macrophage activation,^42,43^ suggesting that upregulation of ROS may promote LNP-cholesterol oxidation. To verify this and further explore the impact of macrophage polarization state on LNP-cholesterol metabolism, we incubated murine bone marrow-derived macrophages (BMDMs) that were unpolarized (M0) or polarized (M1 or M2) with LNPs containing cholesterol-d7 at 97 μM of cholesterol-d7 for 24 h. Then, we collected the cell pellet and supernatant separately for quantitation of LNP-derived cholesterol and oxysterols, as above. The only major metabolite in the cell culture supernatant was 7-KC, and it was found in similar concentrations as the media-only control (Figure 2B), indicating that LNP-cholesterol undergoes auto-oxidation in the cell culture environment. In the cell pellet, we found similar levels of LNP-derived cholesterol in nonactivated and activated macrophages (Figure 2A). However, LNP-derived oxysterols were higher in M0 and M2 macrophages compared to M1 macrophages, with 7-KC, 7β-HC, and 7α-HC as the predominant cholesterol metabolites (Figure 2B–D), while 5β,6β-EC, 24-HC, 25-HC, and 27-HC were not detectable (Supplemental Figure 1), indicating that the primary in vitro oxidation pathway for LNP-cholesterol in macrophages was through auto-oxidation. Contrary to M2 macrophages that are marked by increased fatty acid oxidation and oxidative phosphorylation, inflammatory M1 macrophages express high levels of iNOS, have reduced mitochondrial oxidative capacity, and increased glycolysis, de novo fatty acid synthesis, and accumulation of lipid droplets.^1,44^ Due to increased oxidative stress present in M1 macrophages, we expected to see a higher amount of oxysterols produced by auto-oxidation in these cells. Surprisingly, fewer oxysterols were produced compared to M0 and M2 macrophages. This could have been due to upregulation of antioxidant enzymes, such as peroxiredoxin (peroxidases), an intrinsic self-protection mechanism against oxidative stress in M1 macrophages,^45,46^ which could also affect cholesterol oxidation. Importantly, endogenous oxysterols are known to be potent signaling molecules with heterogeneous effects on immune responses, which are dependent on the oxysterol structure, tissue microenvironment, disease state, and target/receptor (Supplemental Table 2),^47^ but this has not been extensively studied in the context of LNP carriers that are currently used in the clinic.

**Figure 2:**
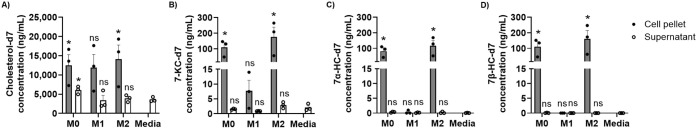
LNP-derived deuterated cholesterol and deuterated oxysterols in murine bone marrow-derived macrophages were quantified by LC–MS/MS.^48^ A) Concentration of cholesterol-d7 and B–D) deuterated oxysterols in cell pellets, supernatants, and media-only control. Data represent mean + SEM; statistical analyses were performed by unpaired *t*-tests compared to media, where * *p* ≤ 0.05. Each data point represents a biological replicate.

### LNPs Modulate Macrophage Immune Functionality and Lipid Homeostasis

Oxysterols act as signaling molecules that influence immune cell activity^49,50^ through various mechanisms, including activation of pattern recognition receptors (PRRs),^47^ stimulation of NLRP3 inflammasome cascades,^51^ induction of ROS,^52^ and binding to nuclear receptors such as liver X receptor (LXR) α/β, retinoic acid receptor-related orphan receptor (ROR) α, RORγt, and estrogen receptor α (ERα).^53^ To determine whether LNP-oxysterols affect macrophage immune functionality, we incubated BMDMs with LNPs containing either cholesterol or one of the oxysterols identified from the above in vivo and in vitro studies (5β,6β-EC, 7-KC, 7α-HC, 7β-HC, 24-HC, and 27-HC) or vehicle control and evaluated gene expression profiles by reverse transcription quantitative polymerase chain reaction (RT-qPCR). All LNP formulations were verified to be monodispersed with similar size and zeta potential (Supplemental Table 1).

We found that 7-KC was the only oxysterol that induced a pronounced proinflammatory transcriptional reprogramming in BMDMs, which was characterized by upregulation of genes for inflammatory cytokines (TNF-α, IL-1β, IL-6) and chemokines (CXCL-10), as well as iNOS (Figure 3A and C). Concurrently, there was suppression of anti-inflammatory genes, including IL-10, ARG-1, and CD206 (Figure 3A and C), suggesting a shift from tissue repair and anti-inflammatory responses toward the maintenance of chronic inflammation. The main effect of the other oxysterols on BMDMs is the suppression of anti-inflammatory markers Mgl-2, CD206, TGF-β, IL-10, and Arg-1, with 7β-HC having the most pronounced effect (Figure 3A–G). These are also markers of M2 macrophages, suggesting the inhibition of immunosuppressive functionality in BMDMs. Notably, VEGF-R2 was upregulated by 7-KC and 7β-HC but not by the other oxysterols. The impact of these oxysterols on lipid metabolism genes showed a trend toward downregulation of de novo fatty acid synthesis (FASN, SREBP1c), mitochondrial β-oxidation (HADHB), and cholesterol transport (APOE) pathways, along with upregulation of LXRα, ABCA1, and RORα, which may promote cholesterol efflux and decrease intracellular cholesterol accumulation.

**Figure 3:**
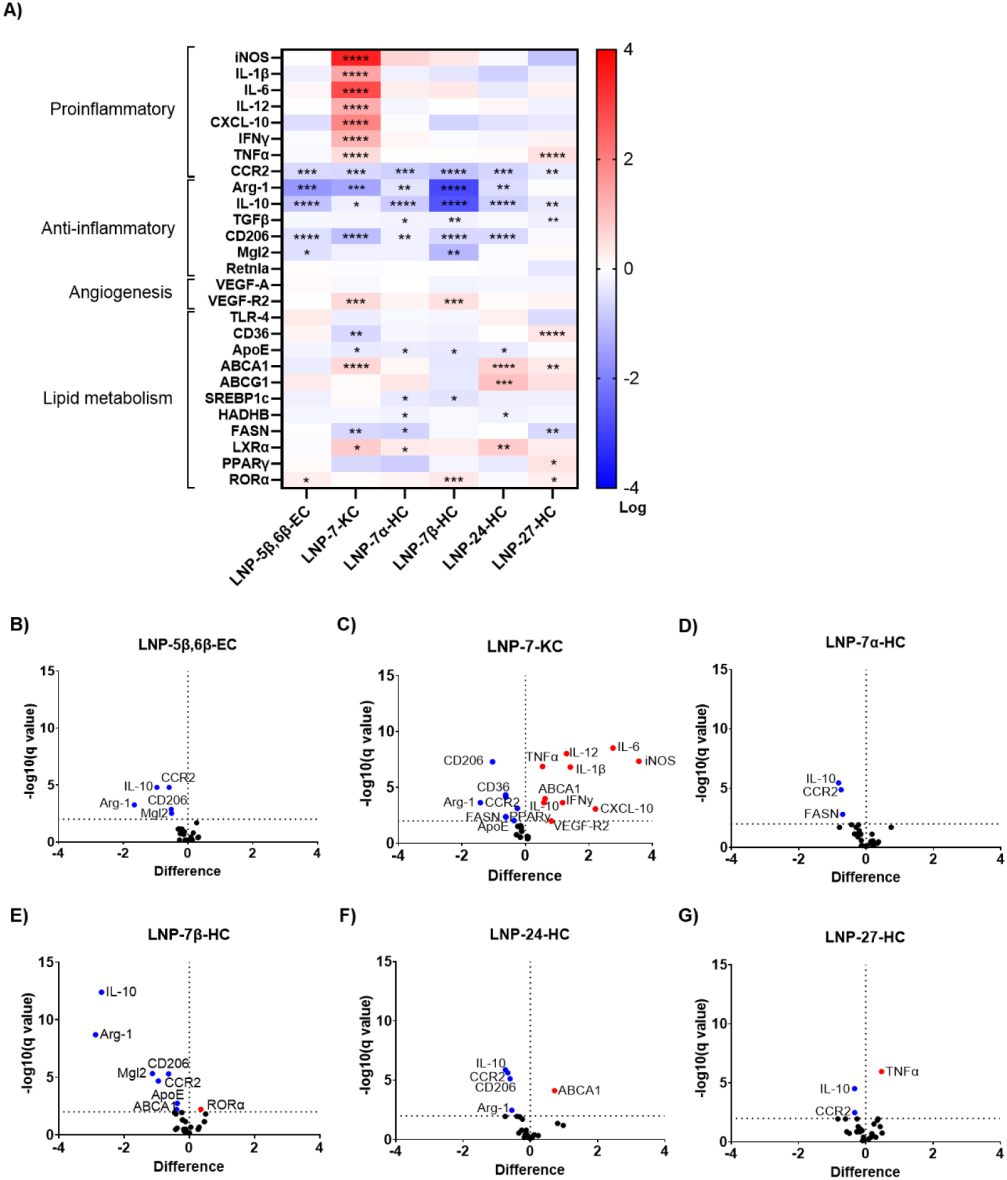
LNP-oxysterols regulate genes involved in inflammation and lipid metabolism in macrophages. A) Heatmap of proinflammatory, anti-inflammatory, angiogenesis, and lipid metabolism genes expressed as log fold-change relative to vehicle. Statistical analyses were performed by one-way ANOVA without correction for multiple comparisons, where * *p* ≤ 0.05; ** *p* ≤ 0.01; *** *p* ≤ 0.001; **** *p* ≤ 0.0001. B–G) Volcano plots against 1% false discovery rate showing upregulated (red) and downregulated (blue) genes compared to vehicle-treated macrophages. Individual bar graphs are in Supplemental Figures 2 and 3.

### LNP-Cholesterol and LNP-Oxysterols Have Heterogeneous Effects on Tumor Growth

To elucidate whether LNP-cholesterol has the potential to impact tumor growth and to dissect the effects of each LNP-oxysterol, wild-type C57BL/6 mice bearing subcutaneously implanted TC-1 tumors were randomized to receive one of these LNP formulations (Supplemental Table 2) at a dose of 47 nmol phospholipids/g (based on the equivalent dose of phospholipids in 8 mg/kg of PLD typically administered in C57BL/6 mice) or vehicle control, and we monitored tumor growth and survival to humane end points. We found that LNP-cholesterol, LNP-24-HC, LNP-27-HC, and LNP-7α-HC had similar moderate but statistically significant inhibition of tumor growth compared to the vehicle control (Figure 4A and B); LNP-7β-HC also significantly inhibited tumor growth (Figure 4A) and was the only oxysterol to decrease the tumor *K*_i_-67 index compared to the vehicle control (Figure 4C and F). Although LNP-7-KC did not show a statistically significant difference in tumor growth compared to vehicle-treated mice, LNP-5β,6β-EC showed a trend suggesting enhancement of tumor growth (Figure 4A and B) and was associated with a significantly increased tumor *K*_i_-67 index (Figure 4D and F), indicating enhanced tumor cell proliferation. To clarify the effects of these two oxysterols, we performed a follow-up survival study and found that both LNP-5β,6β-EC and LNP-7-KC significantly reduced survival rates when compared to LNP-cholesterol (Figure 4E). Together with the quantitative data on LNP-derived oxysterols above, these findings suggest that the antitumoral effect of LNP-cholesterol observed in the TC-1 tumor model (Figure 4A and B) is likely driven by LNP-cholesterol oxidation into 7α-HC, 7β-HC, 27-HC, and 24-HC that we observed in vivo (Figure 1). These results also suggest that 7β-HC and 5β,6β-EC may have direct antitumoral and protumoral effects, respectively, on tumor cell proliferation, whereas the effects of 24-HC, 27-HC, 7α-HC, and 7-KC are primarily due to immune modulatory mechanisms.

**Figure 4:**
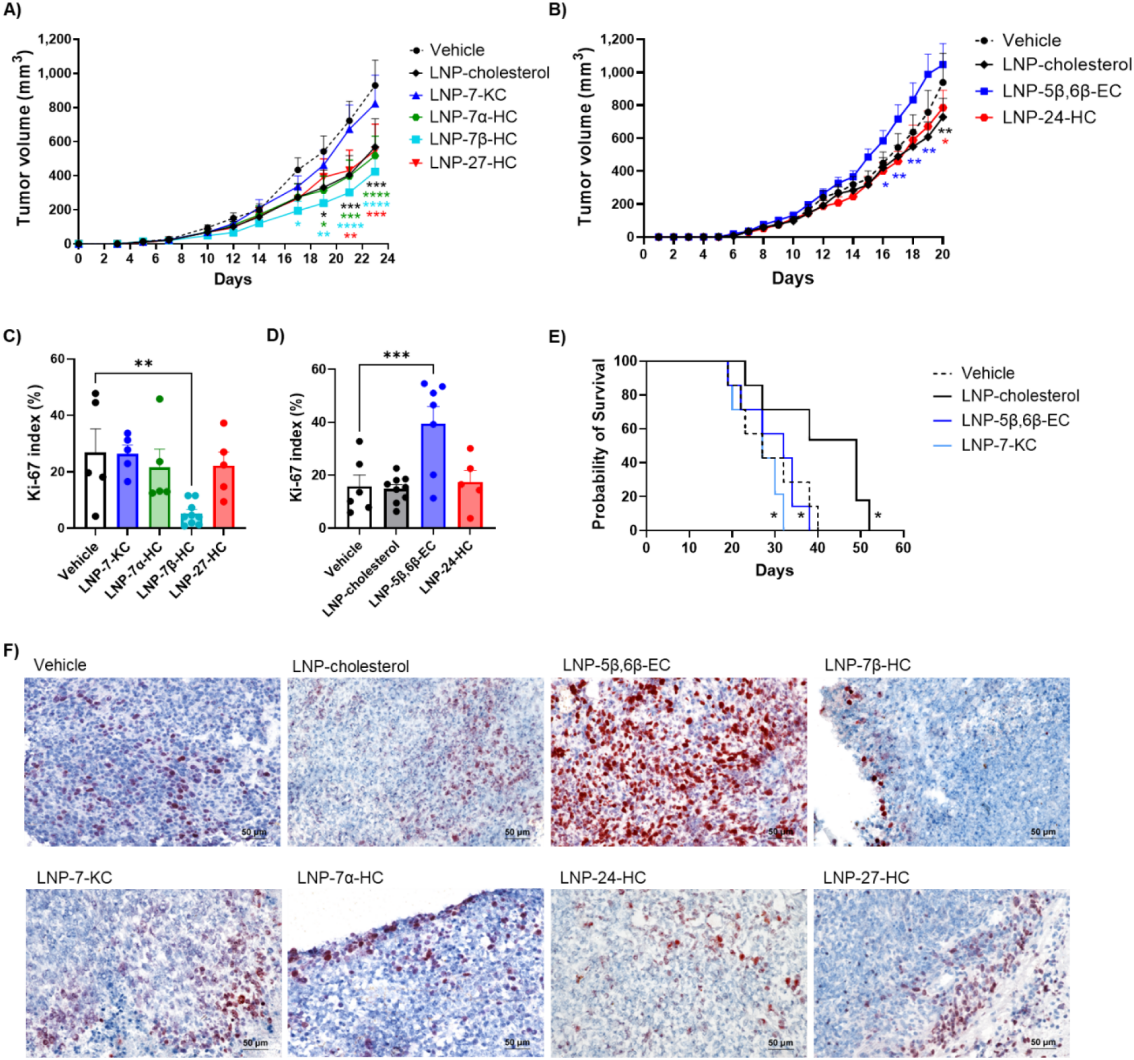
LNP-oxysterols regulate tumor growth in mice bearing TC-1 tumors. A–B) Compared to the vehicle, LNPs containing cholesterol, 24-HC, 7β-HC, 7α-HC, and 27-HC decreased tumor growth, while LNPs containing 5β,6β-EC and 7-KC showed a trend toward increased tumor growth. Data represent mean + SEM, *n* = 10 mice/group, all groups were compared to vehicle by two-way ANOVA without correction for multiple comparisons. C–D) LNPs containing 5β,6β-EC increased the *K*_i_-67 index, while LNPs containing 7β-HC decreased the *K*_i_-67 index. Data represent mean + SEM, all groups were compared to vehicle by one-way ANOVA without correction for multiple comparisons. E) LNPs containing 5β,6β-EC and 7-KC decreased overall survival compared to LNPs containing cholesterol. Kaplan–Meier survival curves with log-rank test comparing 5β,6β-EC LNP and 7-KC LNP groups to cholesterol LNP and cholesterol LNP compared to vehicle, *n* = 7 mice/group. *p < 0.05; **p < 0.01; ***p < 0.001; ****p < 0.0001. F) Representative *K*_i_-67-stained images are shown.

To clarify whether LNP-oxysterols have direct effects on tumor cell proliferation/cytotoxicity, we incubated TC-1 tumor cells with either LNP-cholesterol, one of the LNP-oxysterols, or vehicle control, and then collected cells for MTT analysis of viability and apoptosis and necrosis evaluation by propidium iodide/annexin V staining with FACS analysis. We found that LNP-5β,6β-EC initially induced TC-1 tumor cell proliferation (Figure 5A), but over time, all the LNP-oxysterols induced tumor cell cytotoxicity, with 7β-HC inducing the highest levels of apoptosis and necrosis (Figure 5B–G). These data, together with the in vitro data in BMDM and the in vivo tumor growth data, strongly support that the antitumoral effects of 7β-HC are due to both direct effects on tumor cell proliferation and immune modulatory effects on macrophages. While the cytotoxic effects of 7α-HC, 24-HC, and 27-HC on tumor cells were moderate, these oxysterols significantly decreased anti-inflammatory markers in macrophages, supporting the idea that the antitumoral effects seen in vivo are primarily dependent on immune modulation. Interestingly, while 7-KC was cytotoxic to tumor cells in vitro, it enhanced tumor growth in vivo and induced the expression of proinflammatory genes in macrophages in vitro, suggesting that the protumoral activity of this oxysterol is driven by chronic inflammation within the tumor microenvironment.

**Figure 5:**
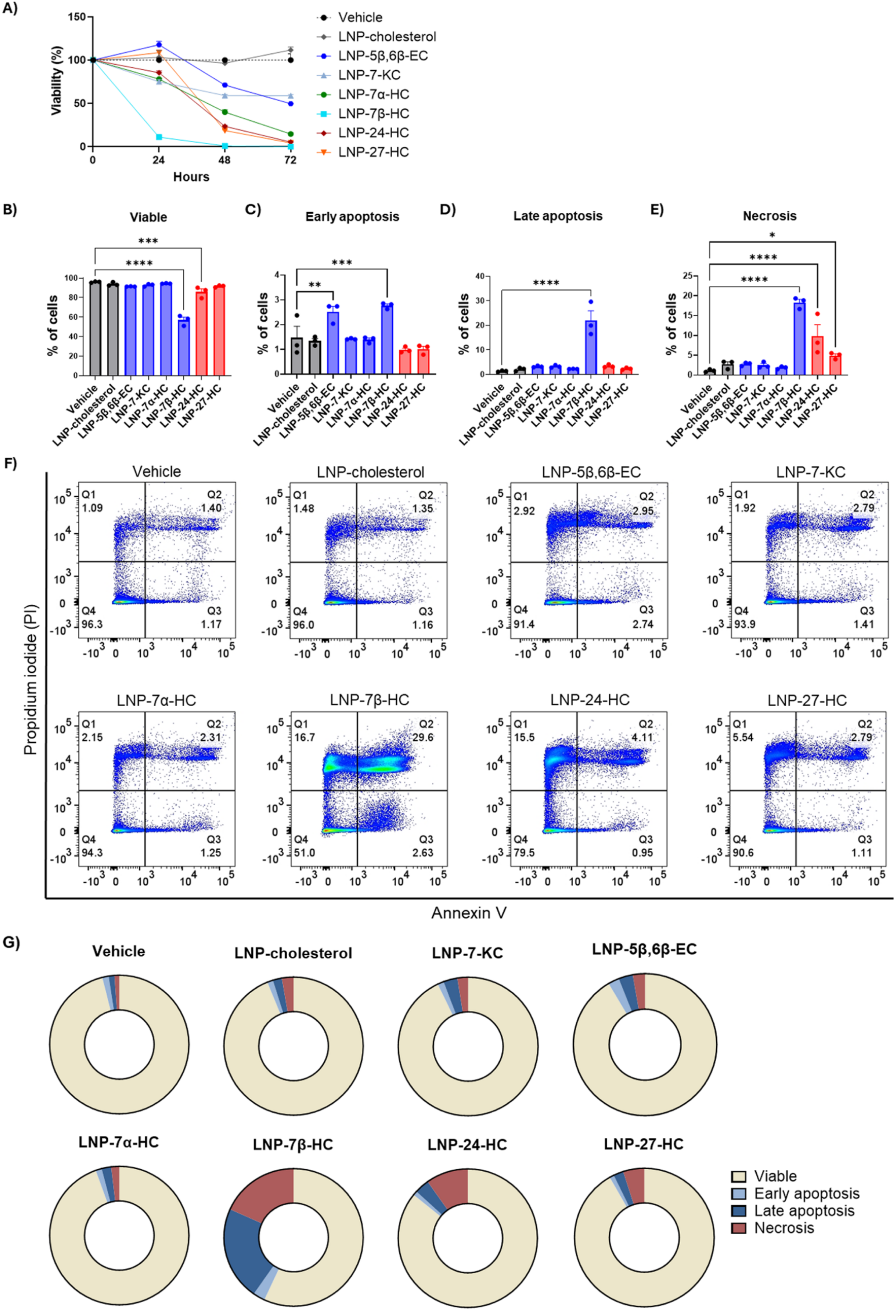
LNP-oxysterols reduce tumor cell viability by inducing late apoptosis and necrosis in vitro. A) TC-1 tumor cell viability at 24, 48, and 72 h by MTT assay. B–E) Apoptosis and necrosis assessed at 24 h by propidium iodide (PI)/annexin V staining. Each data point represents a biological replicate. Statistical tests were performed by one-way ANOVA compared to vehicle without correction for multiple comparisons, where * *p* ≤ 0.05; ** *p* ≤ 0.01; ***p ≤ 0.001; **** *p* ≤ 0.0001. F) Representative flow cytometry plots, where PI^–^/annexin V^–^ are viable cells, PI^+^/annexin V^+^ are cells in late apoptosis, PI^–^/annexin V^+^ are cells in early apoptosis, and PI^+^/annexin V^–^ are cells in necrosis. G) Pie charts of the percentage of viable, early apoptotic, late apoptotic, and necrotic cells.

## Discussion

It is increasingly apparent that enhanced drug delivery alone is not sufficient and that mobilization of an antitumor immune response is necessary for complete tumor eradication in cancer patients. We showed for the first time that a variety of immune-modulatory oxysterols are produced from LNP-cholesterol and that these oxysterols modulate tumor growth at clinically relevant concentrations. Our findings are important because they show that LNP carriers have an unintended impact on tumor growth, which has the potential to diminish or enhance the anticancer efficacy of the LNP-mediated drug. Our study also highlights the potential to engineer LNP carriers with immune-modulatory activity that combines the advantages of both nanoparticle-mediated drug delivery and immunotherapy.

Previously, we reported that a commercial LNP-cholesterol had protumoral effects that were associated with enhanced tumor angiogenesis and immune suppression, which were abrogated with macrophage depletion.^54,55^ When we evaluated the lipid profile of macrophages treated with these commercial LNPs using lipidomics, we found that the total glyceride (TG) level was reduced, while the total phosphocholine (PC) level was increased, consistent with the lipidomic profile of M2 macrophages, whereas M1 macrophages showed increased level of TG and decreased PC (Supplemental Figure 4), suggesting that these commercial LNPs induced M0 macrophages toward an M2-polarization state. In contrast, our current study shows that LNP-cholesterol had antitumoral effects and induced an M1 profile in macrophages. The lipid composition, size, and zeta potential of the two LNP formulations were similar; however, a critical difference is that the liposomes used in the present studies were made before each experiment, manufactured, and stored under inert gas. We theorize that the commercial LNP formulation had undergone significant oxidation during manufacturing/storage, probably generating 7-KC, the predominant oxysterol that we observed forming spontaneously in media, and which we found to be one of the oxysterols with protumoral effects that enhanced the inflammatory phenotype in macrophages.

The LNP-derived oxysterols that we reported here, 7-KC, 7β-HC, 5,6-EC, and 7α-HC, have been found in other commercial LNP preparations at levels up to 7.6 mg/mL of oxysterols,^37,56^ indicating that LNP-associated cholesterol readily undergoes oxidation during manufacturing and/or storage. An important implication of this is that LNP formulations should be monitored for the production of oxidized products and stored protected from light and in an oxygen-free environment to avoid auto-oxidation. However, these solutions would not prevent oxidized products from forming in vivo; antioxidants such as vitamin E could be added to LNP formulations, or cholesterol could be replaced with analogs such as β-sitosterol and cholestanol, which block key oxidation sites.^28,57,58^ Nonetheless, some oxysterols, such as 7β-HC, may have desirable antitumoral and immune-stimulatory effects, and it may not be optimal to block all cholesterol oxidation pathways.

Although we did not look into differences in terms of tumor accumulation and/or LNP uptake efficiency by cancer cells, it is possible that modifications in the LNP sterol group would impact the carrier’s distribution and uptake by affecting stability, protein corona, endosomal escape, and content release.^28,59–62^ In our study, LNP size, zeta potential, phospholipids, and lipid ratios were kept constant among our formulations;^63^ thus, no major changes in tumor accumulation and cell uptake were expected. However, some oxidized cholesterols, such as 25-HC and 20α-HC, have higher delivery to Kupffer cells and endothelial cells in the liver compared to hepatocytes,^62^ suggesting that changes in cellular uptake could occur.

We did not evaluate the impact of LNP-oxysterols on the stable association or premature release of LNP cargo, but modification in the LNP sterol group can alter LNP stability and particle disassembly.^14–17^ LNP-7α-HC has been shown to affect endosomal processing, increasing mRNA delivery to T cells in vitro and ex vivo,^64^ whereas other cholesterol derivatives (e.g., vitamin D derivatives) seem to radically reduce transfection.^28^ Altering the structure of the cholesterol tail tends to increase cell delivery more than modification in the B ring.^62^ C24 alkyl modifications in LNP-cholesterol have been shown to increase mRNA transfection through changes in LNP surface morphology that increase intracellular uptake and membrane destabilization, which increase endosomal escape.^28^ LNP-stigmasterol decreased mRNA encapsulation but maintained transfection efficiency, while LNP-β-sitosterol maintained encapsulation efficiency and increased mRNA transfection in a macrophage cell line.^28^ β-sitosterol induced a polyhedral shape of the LNPs, facilitating endosomal escape and leading to a 200-fold increase in mRNA transfection compared to cholesterol.^61^ Similarly, another study reported that β-sitosterol increased endosomal perturbation events 10-fold compared to cholesterol LNPs.^65^ Contrary to findings in macrophages, complete replacement of cholesterol with β-sitosterol reduced transfection efficiency in a dendritic cell line,^27^ highlighting that the impact of LNP components is cell type-dependent and warrants further investigation. Although the direct consequences of mRNA and an ionizable lipid on cholesterol fate were not the primary focus of this study, these effects are theoretically possible. The ionizable lipid is uncharged at physiological pH, and cholesterol remains tightly associated with the LNP, but the ionizable lipid becomes protonated at a lower pH, increasing electrostatic repulsion. This triggers a phase transition and destabilizes packing, leading to increased exposure of cholesterol,^66^ which can potentially increase cholesterol oxidation in endosomes/lysosomes.

Oxysterols have diverse biological effects in cancer that are dependent on the specific oxysterol species, its receptor affinities, tissue and cellular concentrations, cell-type-specific signaling pathways, the metabolic state and immunologic milieu of the tumor microenvironment, and host factors such as sex hormones and comorbidities.^67–70^ Tumor-derived 27-HC enhanced estrogen receptor-driven tumor growth and LXR-dependent metastatic seeding in murine models of breast cancer,^71^ while in murine models of lymphoma and lung cancers, 27-HC-driven tumor progression was found to be LXR-independent.^21^ In contrast to these studies evaluating tumor-derived oxysterols, we found that LNP-27-HC had moderate antitumor effects in the TC-1 tumor model. It is likely that cholesterol and oxysterols in LNP undergo different intracellular transport and processing than cell-derived cholesterol and oxysterols, which are mostly esterified and associated with lipoproteins, albumin, or cellular membranes. Given that large amounts of ROS are generated in the tumor microenvironment by immune and tumor cells^72^ and that ROS production can be further enhanced by chemotherapies,^73^ it is likely that cholesterol in current commercial liposomal chemotherapy is oxidized in the tumor microenvironment, with the potential to produce more oxysterols than liposomes without any drug cargo. It is also possible that these oxysterols, once produced after initial LNP cellular uptake by macrophages, for example, can be exported into the extracellular fluid in intact nanoparticles or associated with lipid transporters such as LDL and subsequently be internalized by other cells in the tumor microenvironment, leading to additional therapeutic effects.^74^

Unraveling the complex molecular mechanisms that underlie the biological activity of LNP-derived oxysterols will be critical for the development of strategies to leverage cholesterol and its analogs or metabolites to enhance the efficacy of LNP-mediated therapies. In addition, the LNP drug cargo can influence oxysterol generation, and the reverse is also true; oxysterols can influence drug efficacy.^67^ For instance, tamoxifen induced the formation of 5α,6α-EC and 5β,6β-EC in a breast cancer cell line by binding to cholesterol-5,6-hydrolase, changing its activity and generating ROS, with 5,6-EC formation contributing to increased cell sensitivity to tamoxifen.^58^ Similar effects were seen with dendrogenin A, a downstream metabolite of 5α,6α-EC, which promoted tumor suppression in vitro and in vivo, improving the efficacy of cytarabine.^75^ Doxorubicin induced the production of 7β-HC, 7-KC, 7α-HC, 24­(*S*)-HC, and 27-HC in cardiomyocytes, which may be responsible for the cardiotoxicity of doxorubicin. In cancer cells, 4β-HC, 7α-HC, and 27-HC increased the cytotoxicity of doxorubicin in ER-positive cancer cells, while 7-KC decreased efficacy.^76^ In hepatoma cell lines, 7-KC increased P-glycoprotein (P-gp) post-translational expression, which increases drug efflux, thereby decreasing doxorubicin efficacy.^77^ Indeed, multiple cholesterol ABC efflux transporters (e.g., ABCA1 and ABCG1) are also known to transport both oxysterols^78^ and anticancer drugs such as anthracyclines, methotrexate, 5-FU, taxanes, and vinca alkaloids.^79,80^ The modulation of efflux transporters by oxysterols is dependent on cell type and can significantly impact the efficacy of anticancer treatments.

While our study focused on oxidized cholesterol metabolites, LNP-associated phospholipids can also be oxidized.^81,82^ Phospholipid metabolites such as 1-palmitoyl-2-(5-oxovaleroyl)-*sn*-glycero-phosphocholine (POVPC) and 1-palmitoyl-2-glutaroyl-*sn*-glycero-phosphocholine (PGPC) have been linked to cellular stress and metabolic modulation, increased inflammation, autophagy, and epithelial–mesenchymal transition (EMT), leading to increased migration and invasion ability of tumor cells.^81^ This possibility was not explored in our study, but it warrants future investigation to further understand the different effects seen between different LNP formulations.

## Conclusion

We showed that LNP-associated cholesterol is metabolized in vivo into oxysterols that have a heterogeneous impact on tumor growth, tumor cell proliferation, and macrophage functionality. Of these oxysterols, 7-KC and 5β,6β-EC have protumoral effects, raising concerns about possible reductions in the anticancer efficacy and promotion of therapeutic resistance induced by the LNP carrier, whereas 7β-HC has significant antitumoral activity, raising the possibility of designing LNP carriers that enhance the efficacy of the therapeutic cargo. To fully leverage LNPs for cancer therapy, it is imperative to understand the impact of LNP carriers in the context of each therapeutic cargo and tumor model, since the tumor immunologic milieu, the extent to which ROS is generated in the tumor microenvironment, and tumor cell aberrations in cholesterol/oxysterol homeostasis and signaling pathways can alter the biological effects of LNPs.

## Methods

### Key Chemicals

Hydrogenated soy phosphatidylcholine (HSPC), methoxy-polyethylene glycol (PEG2000)-distearoyl-phosphoethanolamine (mPEG_2000_-DSPE), cholesterol, and cholesterol-d7 (catalog no. 700041P) were purchased from Avanti Research (Alabama, USA). 5β,6β-Epoxycholesterol was purchased from Sigma-Aldrich (C2648). 7α-Hydroxycholesterol (Cat. #HY-N7264), 7β-hydroxycholesterol (Cat. #HY-113341), 7-ketocholesterol (Cat. #HY-113342), 24-hydroxycholesterol (Cat. #HY-N2370), and 27-hydroxycholesterol (Cat. #HY-N2371) were obtained from MedChemExpress (Monmouth Junction, NJ, USA). Heat-inactivated fetal bovine serum (HI-FBS) (Cat. #MT35016CV), nonessential amino acid (Cat. #25-025CI), l-glutamine (Cat. #25005CI), and sodium pyruvate (Cat. #25000CI) were purchased from Corning (Corning, NY). IL-4 (Cat. #200-18) was purchased from Shenandoah, while LPS-EB Ultrapure (Cat. #tlrl-3pelps) was purchased from InvivoGen. IMDM (Cat. #12440053) was purchased from Gibco. Optima LC–MS grade acetonitrile (ACN), methanol (MeOH), and formic acid were purchased from Fisher Scientific (Hampton, NH). Isotope-labeled internal standards, including d7 deuterated versions of 5α,6α-epoxycholesterol (Cat. #700047P), 5β,6β-epoxycholesterol (Cat. #700014P), 22­(*S*)-hydroxycholesterol (Cat. #700051), and the d6 form of 27-hydroxycholesterol (Cat. #700059P) were purchased from Avanti Research (Alabaster, AL). The d7 form of 7α-hydroxycholesterol (Cat. #D-4064), 7β-hydroxycholesterol-d7 (Cat. #D-4123), 7-ketocholesterol-d7 (Cat. #D-6045), 24-hydroxycholesterol-d7 (Cat #D-6878), and the d6 form of 25-hydroxycholesterol (Cat. #D-6774) were purchased from CDN Isotopes (Quebec, Canada); 24-hydroxycholesterol-d4 (Cat. #TRC-H918042) was purchased from LGC Standards Ltd. (UK).

### Formulations

Liposomes containing 55% HSPC, 5% mPEG_2000_DSPE, and 40% (molar ratio) cholesterol, cholesterol-d7, or oxysterols were synthesized. Briefly, lipids were solubilized and homogenized in a round-bottom flask containing chloroform, then transferred to a rotary evaporator for lipid thin-film formation. To evaporate the remaining solvent, flasks were kept overnight in a vacuum desiccator. The dried film was hydrated using 0.9% sodium chloride, sonicated for 5 min at 60 °C, and shaken for 60 min at 60 °C and 225 rpm. The formulations were then extruded using 0.05 to 1.0 μm membranes with a Lipex liposome extruder from Evonik Industries (Essen, Germany) and a Mini Extruder system from Avanti Polar Lipids (Alabaster, AL, USA). Particle size, polydispersity index (PDI), concentration, and zeta potential were measured using a Zetasizer Ultra from Malvern Panalytical (Malvern, Worcestershire, UK). Liposomes were sterilized using 0.22 μm PES syringe filters. Phospholipid concentration was determined using a modified Rouser Assay^83^ with a standard curve prepared using monobasic sodium phosphate (Sigma-Aldrich, USA) and included quality controls.

### Cell Culture

Bone marrow–derived macrophages (BMDMs) were differentiated from the femur and tibia of C57BL/6 mice using DMEM 10-17-CV supplemented with 20% premium HI-FBS, 30% L929 cell supernatant, 1% l-glutamine, 1% sodium pyruvate, and 1% penicillin/streptomycin. The cells were incubated at 37 °C and 5% CO_2_ for 8 days, with half of the media replaced on day 4. Cells were then replated for experiments using IMDM media supplemented with 10% premium HI-FBS and 1% penicillin/streptomycin. Polarization was performed using 100 ng/mL lipopolysaccharide (LPS) for M1 macrophages and 20 ng/mL IL-4 for M2 macrophages. The cells were incubated with polarization stimuli for 12 h prior to liposomal treatment.

TC-1 tumor cells were cultured at 37 °C/5% CO_2_ using RPMI 1640 supplemented with 10% HI-FBS, 10 mM HEPES, 1 mM sodium pyruvate, 1% nonessential amino acids, and 1% penicillin/streptomycin. Cells tested negative for mycoplasma prior to experiments (Myco-Sniff Mycoplasma PCR Detection Kit, Cat. No. 3050201, MP Biomedicals, Santa Ana, CA, USA).

### In Vitro Studies

To evaluate LNP-cholesterol metabolism in macrophages, 5 × 10^6^ BMDMs were plated in 100 mm^2^ Petri dishes with 10 mL of supplemented IMDM and polarized to the M1 or M2 phenotype, then incubated with LNP-cholesterol-d7 at 167 μM phospholipids (97 μM cholesterol-d7) for 24 h. Cells and supernatants were collected for LC–MS analyses to evaluate the production of deuterated oxysterols. Controls included cells treated with saline and LNP in media without cells. The LNP concentration was based on the maximum plasma concentration of phospholipids reported in patients treated with pegylated liposomal doxorubicin (PLD, also known as Doxil).^31^

For proliferation studies, TC-1 tumor cells or BMDMs were plated in triplicate in 96-well plates, incubated at 37 °C with 5% CO_2_ overnight for acclimation, and then treated with 55.7 μM LNP-cholesterol or LNP-oxysterols for 24, 48, and 72 h. Cytotoxicity and cell proliferation were assessed using the MTT assay kit (Cat. #4890050K, R&D Systems), and absorbance at 570 nm was measured using a Cytation 5 plate reader.

To assess apoptosis and necrosis, TC-1 cells were plated and treated with LNPs as above, then the cells were collected, washed, and resuspended in Annexin V Binding Buffer (catalog no. 422201, BioLegend). Approximately 1 × 10^6^ cells were stained with APC Annexin V (Cat. #640920, BioLegend) and propidium iodide (Cat. #421301, BioLegend). Apoptosis and necrosis controls were used to set up the gating strategies. LSR Fortessa flow cytometer (BD Biosciences, San Jose, CA, USA) and FlowJo software (Tree Star Inc., Ashland, OR, USA) were used for analyses.

To evaluate the immune response induced by LNP-oxysterols, BMDMs were treated in triplicate for 24 h with LNP-oxysterols or vehicle control. Cells were collected with RLT Plus buffer containing β-mercaptoethanol for RT-qPCR analyses of genes associated with macrophage M1 and M2 functionality, and lipid metabolism (Supplemental Table 3). The mRNA was extracted and purified using a QIAGEN RNaesy Plus Mini Kit (Cat. no. 74134), measured by NanoDrop 2000, reversed into cDNA using a High-Capacity cDNA Reverse Transcription Kit with RNase Inhibitor (Cat. no. 4374967), and amplified using real-time quantitative PCR with PowerUp SYBR Green Master Mix, primer mixes (8 μM), and Step One Plus (Applied Biosystems) following 40 amplification cycles. Relative mRNA expression was normalized to GAPDH and calculated using the 2^ΔΔCt^ method and RT^2^ Profiler PCR Array Data Analysis (QIAGEN).

### Animals

Six- to eight-week-old female and male C57/BL6 mice were purchased from Jackson Laboratory (Bar Harbor, ME, USA) and acclimated for at least 1 week at the Texas Tech University Health Sciences Center (TTUHSC) animal care facility (Abilene, TX, USA), according to the Institutional Animal Care and Use Committee (IACUC) guidelines. All experiments were performed under an IACUC-reviewed and approved protocol (TTUHSC, approval number 11005).

To evaluate LNP-cholesterol metabolism in vivo, a total of 100.5 nmol/g of cholesterol-d7 (141.5 nmol/g of phospholipids) was administered to each mouse via a tail vein injection. This dose corresponds to the amount of cholesterol present in the pegylated liposomal doxorubicin (PLD) administered to humans at a dose of 60 mg/m^2^ of doxorubicin^31^ calculated using the human–mouse equivalent dose.^32^ Mice were euthanized with CO_2_ followed by cervical dislocation 24 h post-treatment, and blood was collected in EDTA-treated tubes through cardiac puncture. Blood samples were centrifuged for 10 min at 1,000*g* at 4 °C to obtain plasma. Liver, spleen, heart, kidneys, and lungs were collected, weighed, flash-frozen, then stored at −80 °C along with plasma samples until LC–MS/MS analyses.

For tumor growth studies, 0.5 × 10^6^ TC-1 cells were subcutaneously implanted in the left flanks of female mice. Animals (*n* = 10 mice/group) received LNP-oxysterol or LNP-cholesterol at a dose of 47 nmol of phospholipids/g body weight, or saline vehicle control, every 3 days starting 2 days after implantation until the study end point was reached. This dose was chosen based on an equivalent dose of phospholipids in 8 mg/kg of PLD that is typically administered in C57BL/6 mice.^55,84^ Tumor growth was monitored with a digital caliper, and tumor volume was estimated using volume = (*A*× *B* ^2^)/2, where *A* = largest diameter and *B* = smaller diameter. Animal weight was monitored for signs of systemic toxicity. Tumor tissue was collected at the end point and processed for immunohistochemistry.

For tumor survival studies, treatments were given 1 day post-TC-1 tumor implantation (*n* = 7 mice/group). Mice received vehicle control, LNP-oxysterols, or LNP-cholesterol as described above, for a total of four doses, administered every 4 days. Animals were monitored, and tumors were measured as described above until tumors reached 1000 mm^3^ at which they were euthanized.

### Immunohistochemistry

Tumor tissues embedded in OCT blocks and frozen blocks were sectioned at 5 μm thickness, mounted on charged slides (Cat. #1358W, Globe Scientific), and fixed with acetone at −20 °C. Tissues were blocked with 2% goat serum for 30 min at room temperature, followed by streptavidin and biotin blockage (Cat #SP-2002, Vector Laboratories). Antibody against *K*_i_-67 (Cat. #12202, Cell Signaling) was diluted 1:400 with 1% BSA, 0.5% Triton X-100, and 1× PBS and incubated on tissue sections overnight at 4 °C. Endogenous peroxidase was blocked with 3% hydrogen peroxide for 30 min at room temperature, followed by biotin-conjugated secondary goat antirabbit antibody diluted 1:250 (Cat. #50-194-1796, Jackson Immuno Research Laboratories) in 0.05% Tween 20 and 1× PBS, VECTASTAIN ABC (Cat. #PK-4000, Vector Laboratories) and AEC (Cat. #SK-4205, Vector Laboratories) staining according to manufacturer instructions. Tissues were counterstained with hematoxylin and mounted with aqueous mounting media (Cat. #H-5501, Vector Laboratories). The *K*_i_-67 index was calculated based on the AEC-positive area divided by the hematoxylin-positive area × 100.^85^ A total of 5 highly positive images for *K*_i_-67 were taken at 20× and analyzed using a Nikon AX R confocal.

### Oxysterol Quantification

Deuterated cholesterol and oxysterols were quantified in tissues, plasma, cell pellets, and supernatants following a previously published method for liquid chromatography coupled with tandem mass spectrometry.^48^ A 150 mm C18 column (Acquity Premier Vanguard FITBEH 1.7 μm, 150 × 2.1 mm C18) was maintained at 25°C, and an ACN/water gradient mobile phase system was used. Samples were lysed using Pierce IP lysis buffer supplemented with butylated hydroxytoluene (BHT) and protease inhibitors dissolved in DMSO.

### Statistical Analyses

Two-way ANOVA was used for tumor growth analyses, while one-way ANOVA was used for in vitro analyses. Results were compared to vehicles and were not corrected for multiple comparisons analyses. Survival analyses were performed by using the log-rank test. Volcano plots were generated using multiple *t*-tests with a 1% false discovery rate. Statistical significance was considered for a *p*-value of less than 0.05. All statistical analyses were performed using GraphPad Prism software, version 10 or higher.

## Supplementary Material



## Notes

Supplemental Table 1: liposome characterization; Supplemental Table 2: oxysterols’ effects on immune cells; Supplemental Figure 1: LC–MS/MS analyses of deuterated oxysterols showed that 5β,6β-EC-d7, 24-HC-d7, 25-HC-d6, and 27-HC-d6 were not detectable in bone-marrow-derived macrophages treated in vitrowith liposomes containing D7-cholesterol; Supplemental Figure 2: individual bar graphs for genes involved in immune modulation and angiogenesis; Supplemental Figure 3: individual bar graphs for genes involved in lipid metabolism; Supplemental Figure 4: LNP-cholesterol alters the lipid profile in macrophages; Supplemental Table 3: primers utilized for RT-qPCR analyses (PDF)
