# Supplementary material for: Immune Modulatory Oxysterols Produced from Cholesterol-Containing Lipid Nanoparticles Regulate Tumor Growth
Source: ACS Nano. 2026 Mar 13;20(12):9983–95. doi: 10.1021/acsnano.5c22020 (PMC13045349; doi:10.1021/acsnano.5c22020)
Supplement: Supplementary file 1 [file nn5c22020_si_001.pdf]

Immune Modulatory Oxysterols Produced From Cholesterol-Containing Lipid Nanoparticles Regulate Tumor Growth

Authors: Patricia Ines Back,[1*] Shadan Modaresahmadi,[1*] Jalpa Patel,[1] Indhumathy Subramaniyan,[2,3] Vindhya Edpuganti,[2,3] Md. Rakibul Islam,[1] Seonjuti Chowdhury,[2,3] Adam J. Carr,[4] Qisheng Zhang,[4] William C. Putnam,[2,3] Li Li,[2,3] Ninh M. La-Beck[1]

[1] Department of Immunotherapeutics and Biotechnology, Jerry H. Hodge School of Pharmacy, Texas Tech University Health Sciences Center, Abilene, TX 79601, USA

[2] Clinical Pharmacology and Experimental Therapeutics Center, Jerry H. Hodge School of Pharmacy, Texas Tech University Health Sciences Center, Dallas, TX, USA

[3] Department of Pharmacy Practice, Jerry H. Hodge School of Pharmacy, Texas Tech University Health Sciences Center, Dallas, TX 75235, USA

[4] Division of Chemical Biology and Medicinal Chemistry, Eshelman School of Pharmacy, University of North Carolina at Chapel Hill, Chapel Hill, NC 27599, USA

* Authors contributed equally to this work

Correspondence:

Ninh M. La-Beck (irene.la-beck@ttuhsc.edu), Department of Immunotherapeutics and Biotechnology, Jerry H. Hodge School of Pharmacy, Texas Tech University Health Sciences Center, 1718 Pine St, Abilene, Texas 79601, USA; telephone 1-325-696-0433

Supplementary Materials

Supplemental Table 1. Liposome characterization.

Formulation	Size (nm)	PDI	Zeta Potential (mV)	Phospholipid concentration (mM)
LNP-cholesterol-d7	92.24 ± 1.83	0.03 ± 0.01	-35.42 ± 1.11	24.53 ± 1.99
LNP-cholesterol	90.73 ± 2.06	0.06 ± 0.02	-35.33 ± 3.28	19.63 ± 2.10
LNP-5β,6β-epoxycholesterol	90.42 ± 3.74	0.05 ± 0.03	-34.83 ± 1.18	17.66 ± 1.90
LNP-7-ketocholesterol	94.74 ± 5.44	0.04 ± 0.01	-35.32 ± 3.54	14.63 ± 2.88
LNP-7β-hydroxycholesterol	86.01 ± 2.44	0.09 ± 0.02	-33.91 ± 3.46	18.17 ± 2.40
LNP-7α-hydroxycholesterol	91.46 ± 3.18	0.07 ± 0.02	-33.36 ± 3.08	17.48 ± 1.42
LNP-24-hydroxycholesterol	91.44 ± 2.28	0.11 ± 0.03	-33.17 ± 2.66	15.83 ± 2.44
LNP-27-hydroxycholesterol	82.22 ± 5.19	0.14 ± 0.04	-33.39 ± 2.26	14.88 ± 0.85

Supplemental Table 2. Oxysterols effects on immune cells.

Oxysterol	Primary targets/MOA	Immune Effects
5,6-Epoxycholesterol (5,6-EC)[1]	Increases protein kinase C (PKC) activity, enhances superoxide anion release (ROS), activates NADPH oxidase, and caspase-2L	Increases M1 macrophages, proinflammatory cytokines (TNF-α, IL-1β, and IL-6) production, oxidative stress in macrophages, LDL oxidation resulting in chronic inflammation, and apoptosis in leukemia cell lines (U937 and HL-60)
7-Ketocholesterol (7-KC)[1]	Agonist of RORα and ERα, activates proinflammatory genes<br><br>Induces ROS generation leading to protein/lipid/DNA damage<br><br>LXR agonist, but proinflammatory effects are LXR-independent	Strongly proinflammatory via cytokines/chemokine induction<br><br>Promotes foam cell formation<br><br>Causes ROS-mediated apoptosis of macrophages, endothelial, and smooth muscle cells<br><br>Upregulates LXR-targeted genes (ABCA1, ABCG1, and SREBP-1c), but remains proinflammatory
7α-Hydroxycholesterol (7α-HC)[2-4]	Suppresses transcriptional activity of ROR	Induces myeloid cell maturation into dendritic cells<br><br>Enhances macrophage secretion of IL-8 (CXCL8) and CCR5 ligands (CCL3/CCL-4)
7β-Hydroxycholesterol (7β-HC)[2-4]	Suppresses transcriptional activity of ROR<br><br>Induces ROS, resulting in oxidative stress<br><br>Activates inflammatory signaling pathways	Increases the secretion of IL-1β<br><br>Enhances the expression of adhesion molecules, leading to inflammation and the recruitment of immune cells<br><br>Increases proinflammatory cytokines, including MCP-1, TNFα, IL-1β, and IL-8 in monocytes<br><br>Induces oxidative stress and apoptosis via ROS in macrophages
24-Hydroxycholesterol (24-HC)[2-5]	Inverse agonist of RORα/γ; suppresses ROR-driven transcription<br><br>Activates LXR, resulting in cholesterol efflux and reduced TLR signaling<br><br>Activates SIRT1 by altering redox state, resulting in deacetylation and suppression of inflammatory genes	Anti-inflammatory in macrophages<br><br>Decreases the production of TLR-mediated cytokines<br><br>In microglia, SIRT1 activation represses inflammatory genes
27-Hydroxycholesterol (27-HC)[1, 5]	Activates LXR, induces cholesterol efflux genes, and regulates lipid metabolism<br><br>Agonist of RORα/γ and activates Th17-related genes<br><br>Binds to RORγt and causes the promotion of IL-17-producing cells	Anti-inflammatory effect via LXR, which reduces M1 markers (CD80/CD86) and increases M2 markers (CD163/CD206)<br><br>Enhance proinflammatory cytokines via ROR pathways<br><br>Increases the expression of IL-10, CCL-2, LXR, and ABCA1<br><br>Triggers secretion of CCL3/CCL4 in monocytic cells and enhances Th1 cell migration

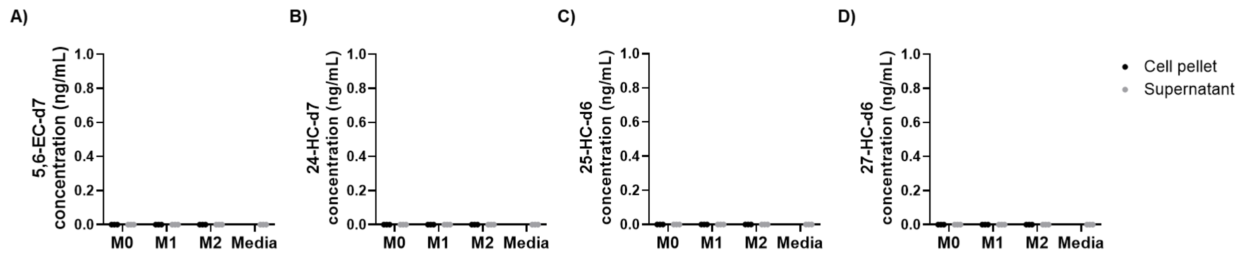

Supplemental Figure 1. LC-MS/MS analyses of deuterated oxysterols showed that 5β,6β-EC-d7, 24-HC-d7, 25-HC-d6, and 27-HC-d6 were not detectable in bone-marrow derived macrophages treated *in vitro* with liposomes containing deuterated cholesterol (LNP-cholesterol-d7).

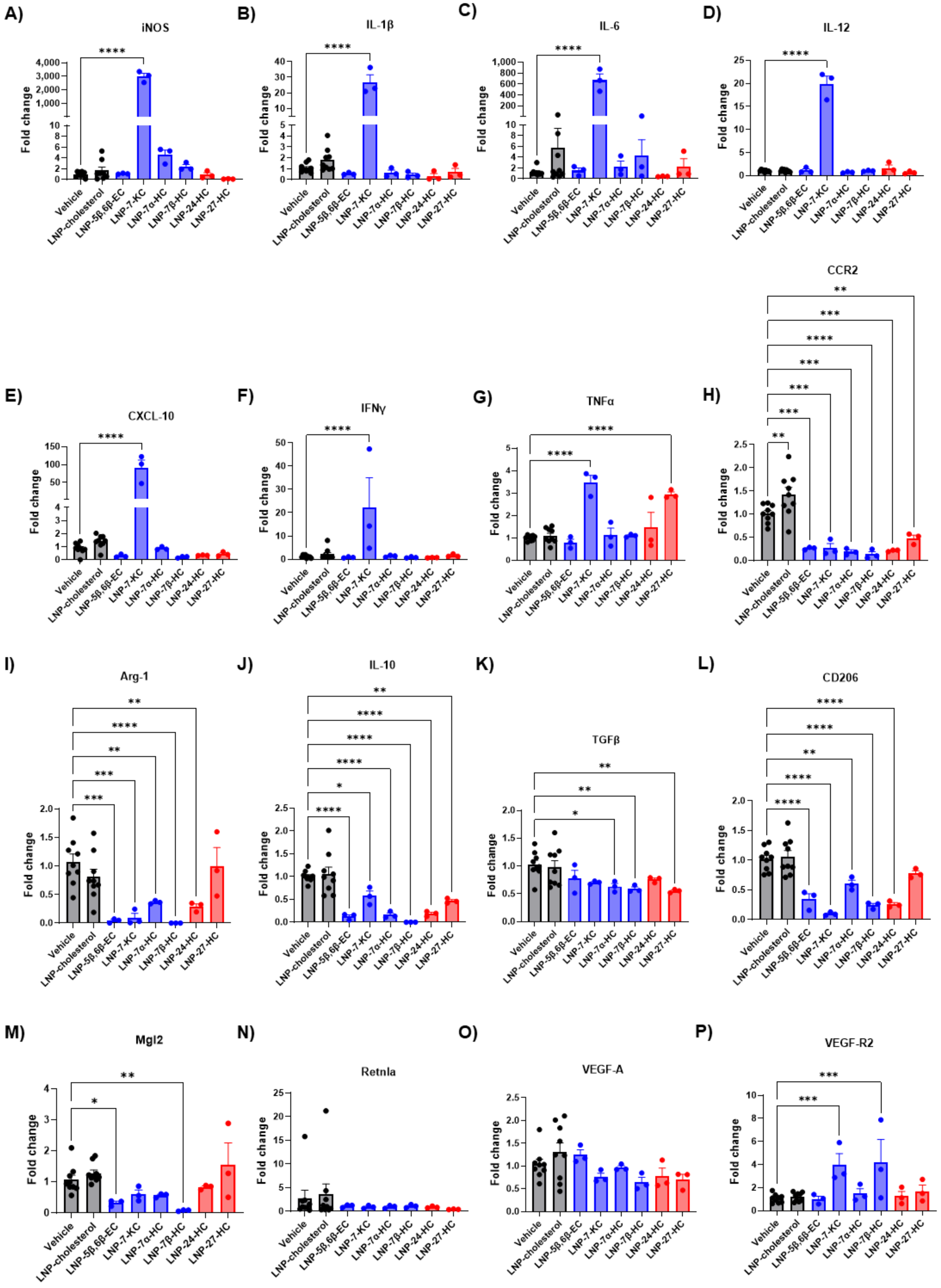
A)
iNOS
B)
IL-1β
C)
IL-6
D)
IL-12
E)
CXCL-10
F)
IFNγ
G)
TNFα
H)
CCR2
I)
Arg-1
J)
IL-10
K)
TGFβ
L)
CD206
M)
Mgl2
N)
Retnla
O)
VEGF-A
P)
VEGF-R2
Fold change
Vehicle
LNP-cholesterol
LNP-5β,6β-EC
LNP-7-KC
LNP-7α-HC
LNP-7β-HC
LNP-24-HC
LNP-27-HC

Supplemental Figure 2. LNP-oxysterols modulate immune response in macrophages. A-H) LNP-7-KC increased pro-inflammatory genes expression in BMDMs, while LNP-27-HC upregulated TNFα along with 7-KC. I-N) LNP-oxysterols impacted anti-inflammatory genes expression. O-P) LNP-oxysterols induced expression of VEGF-R2 in BMDMs, while no effects on VEGF-A expression were found. Each data point represents a biological replication. Bars represent mean + SEM. Statistical tests were performed by One-Way ANOVA compared to vehicle, not corrected for multiple comparisons, where * $p \leq 0.05$; ** $p \leq 0.01$; *** $p \leq 0.001$; **** $p \leq 0.0001$.

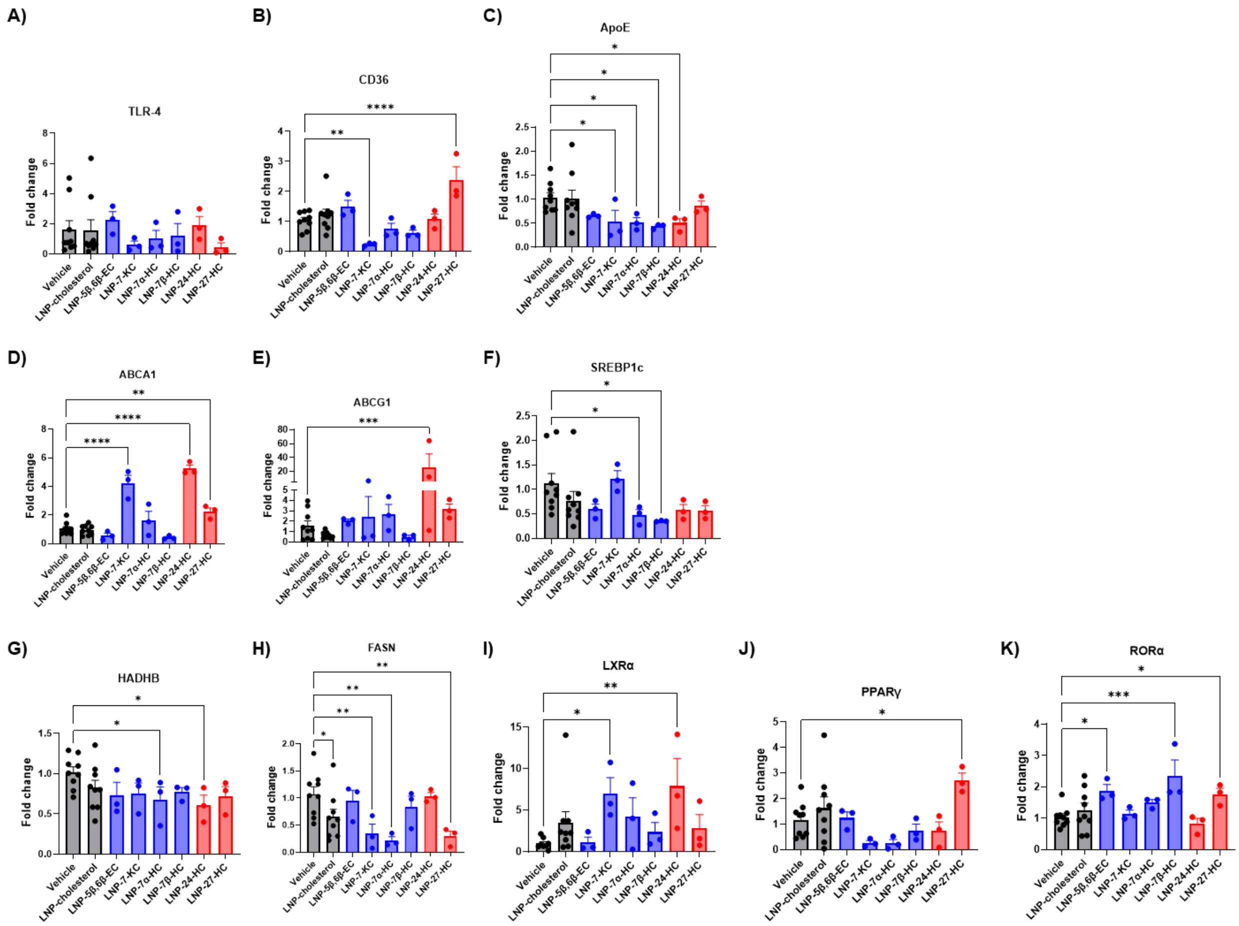

Supplemental Figure 3. LNP-associated oxysterols interfere in genes involved in lipid metabolism. A-C) Represent genes related to liposomal interaction with macrophages; D-E) Represent cholesterol and oxysterols efflux transporters; F-K) Represent genes related to lipid metabolism in macrophages. Each data point represents a biological replication. Bars represent mean + SEM. Statistical tests were performed by One-Way ANOVA compared to vehicle, not corrected for multiple comparisons, where * $p \leq 0.05$; ** $p \leq 0.01$; *** $p \leq 0.001$; **** $p \leq 0.0001$.

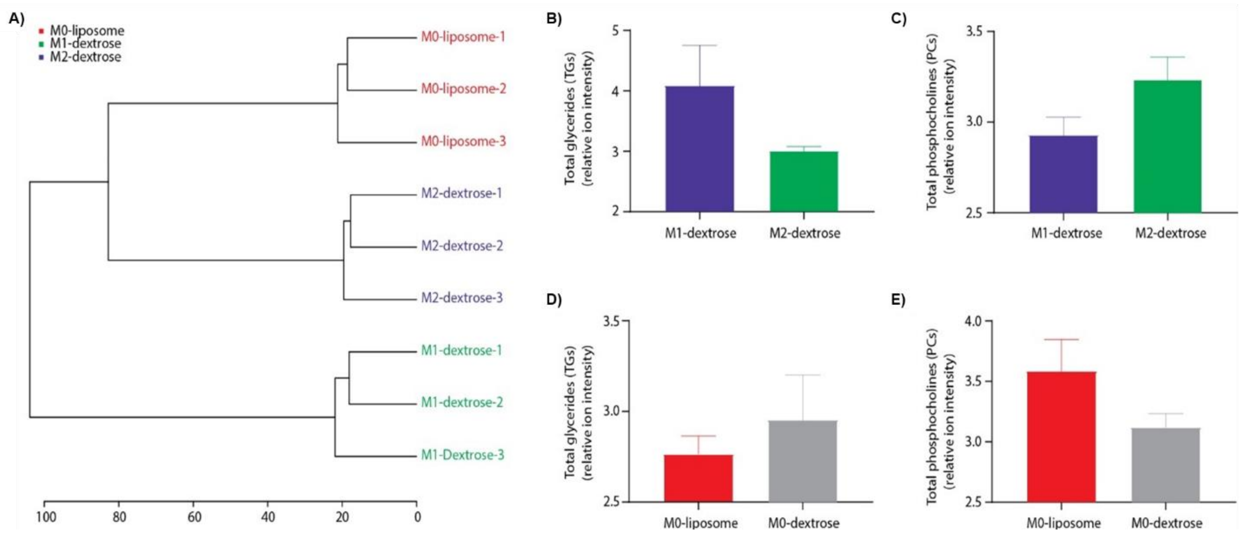

Supplemental Figure 4. LNP-cholesterol modifies lipid profile in macrophages. The lipidome of unpolarized (M0) macrophages treated with a commercial liposome was compared to M1 or M2 polarized macrophages. Bars represent average of 4 replicates. Experiments and lipidomics were performed as described below in supplemental methods.

Supplemental Table 3. Primers utilized for RT-qPCR analyses.

Gene	Forward primer 5'-3'	Reverse primer 5'-3'
iNOS	GTTCTCAGCCCAACAATACAAGA	GTGGACGGGTCGATGTCAC
IL-1β	TGGACCTTCCAGGATGAGGACA	GTTCATCTCGGAGCCTGTAGTG
IL-6	GCTACCAAACTGGATATAATCAGGAAA	CTTGTTATCTTTTAAGTTGTTCTTCATGTACTC
IL-12	ACGAGAGTTGCCTGGCTACTAG	CCTCATAGATGCTACCAAGGCAC
CXCL-10	CCTATGGCCCTCATTCTCAC	CTCATCCTGCTGGGTCTGAG
IFNγ	CAGCAACAGCAAGGCGAAAAAGG	TTTCCGCTTCCTGAGGCTGGAT
TNFα	GGTGCCTATGTCTCAGCCTCTT	GCCATAGAACTGATGAGAGGGAG
CCR2	GCTGTGTTTGCCTCTCTACCAG	CAAGTAGAGGCAGGATCAGGCT
Arg-1	ACCTGGCCTTTGTTGATGTCCCTA	AGAGATGCTTCCAACTGCCAGACT
IL-10	CGGGAAGACAATAACTGCACCC	CGGTTAGCAGTATGTTGTCCAGC
TGFβ	TGATACGCCTGAGTGGCTGTCT	CACAAGAGCAGTGAGCGCTGAA
CD206	GTTCACCTGGAGTGATGGTTCTC	AGGACATGCCAGGGTCACCTTT
Mgl-2	CGAGACTTGAGCCAGAAGGTGA	GCCTTCAAGTCTGTCTCCAGCT
Retnla	CAAGGAACTTCTTGCCAATCCAG	CCAAGATCCACAGGCAAAGCCA
VEGF-A	CTGCTGTAACGATGAAGCCCTG	GCTGTAGGAAGCTCATCTCTCC
VEGF-R2	CGAGACCATTGAAGTGACTTGCC	TTCCTCACCCTGCGGATAGTCA
TLR-4	AGCTTCTCCAATTTTTCAGAACTTC	TGAGAGGTGGTGTAAGCCATGC
CD36	GGACATTGAGATTCTTTTCCTCTG	GCAAAGGCATTGGCTGGAAGAAC
APOE	GAACCGCTTCTGGGATTACCTG	GCCTTTACTTCCGTCATAGTGTC
ABCA1	CCCAGAGCAAAAAGCGACTC	GGTCATCATCACTTTGGTCCTTG
ABCG1	TCGGACGCTGTGCGTTTT	CCCACAAATGTCGCAACCT
SREBP1c	CGACTACATCCGCTTCTTGCAG	CCTCCATAGACACATCTGTGCC
HADHB	GATGGAGGCCAGTATGCTTT	AGTCGGTCGCCTCCTTCTA
FASN	GGAGGTGGTGATAGCCGGTAT	TGGGTAATCCATAGAGCCCAG
LXRα	ATCGCCTTGCTGAAGACCTCTG	CTGCTTTGGCAAAGTCTTCCCG
PPARγ	GTACTGTCGGTTTCAGAAGTGCC	ATCTCCGCCAACAGCTTCTCCT
RORα	CAGAGCAATGCCACCTACTCCT	CTGCTTCTTGGACATCCGACCA
GAPDH	GGTGCTGAGTATGTCGTGGA	GTGGTTCACACCCATCACAA

Supplemental Methods: Macrophage Lipidomics

A total of 2.3x10^6 bone marrow-derived macrophages (BMDMs) was used for each sample; cells were treated with LNP-cholesterol or vehicle for 24 hours. LNP concentration was based on total phospholipid concentration found in Cmax of patients who receive Doxil at 20 mg/m^2 (55.7 μM). To extract lipids, methanol (0.3 mL) was added to a 2 mL centrifuge tube containing cell pellets and the resulting mixture was vortexed for 10 seconds. Methyl tert-butyl ether (MTBE, 1 mL) was then added, vortexed for 10 seconds, followed by addition of water (0.25 mL) and another 10 seconds of vortex. The mixture was centrifuged at 2,000 rcf for 10 minutes at room temperature. The top layer (MTBE) was transferred to a new 2 mL centrifuge tube while the bottom layer was re-extracted. The re-extraction solution was composed of a mixture of 10:3:2.5 MTBE:MeOH:H_2O. The mixture was vortexed for 30 seconds and phase separation was given time to occur. The top layer was then used as the re-extraction solution and added (0.6 mL) to the original centrifuge tube containing the cell pellets. Again, the mixture was vortexed for 10 seconds and left on a shaker at room temperature for 10 minutes, when it was centrifuged at 2,000 rcf for 10 minutes. The top layer (MTBE) was transferred to the previous centrifuge tube containing the first MTBE extraction. The combined mixture was dried, and the lipids were resuspended in isopropanol and analyzed by LC-MS (Waters Acquity H-class UPLC with BEH RP-C18 column, coupled to ThermoScientific Q Exactive HF-X Orbitrap mass spectrometer). LipidSearch was used to identify lipid species for further analysis using MetaboAnalyst 5.0.

References

1. Back, P. I.; Yu, M.; Modaresahmadi, S.; Hajimirzaei, S.; Zhang, Q.; Islam, M. R.; Schwendeman, A. A.; La-Beck, N. M., Immune implications of cholesterol-containing lipid nanoparticles. *ACS nano* **2024,** *18* (42), 28480-28501.

2. Wang, Y.; Kumar, N.; Crumbley, C.; Griffin, P. R.; Burris, T. P., A second class of nuclear receptors for oxysterols: Regulation of RORα and RORγ activity by 24S-hydroxycholesterol (cerebrosterol). *Biochimica et Biophysica Acta (BBA)-Molecular and Cell Biology of Lipids* **2010,** *1801* (8), 917-923.

3. Wang, Y.; Kumar, N.; Solt, L. A.; Richardson, T. I.; Helvering, L. M.; Crumbley, C.; Garcia-Ordonez, R. D.; Stayrook, K. R.; Zhang, X.; Novick, S., Modulation of retinoic acid receptor-related orphan receptor α and γ activity by 7-oxygenated sterol ligands. *Journal of Biological Chemistry* **2010,** *285* (7), 5013-5025.

4. Choi, C.; Finlay, D. K., Diverse immunoregulatory roles of oxysterols—the oxidized cholesterol metabolites. *Metabolites* **2020,** *10* (10), 384.

5. Yanagisawa, R.; He, C.; Asai, A.; Hellwig, M.; Henle, T.; Toda, M., The impacts of cholesterol, oxysterols, and cholesterol lowering dietary compounds on the immune system. *International Journal of Molecular Sciences* **2022,** *23* (20), 12236.
